# Securitizing HIV/AIDS: a game changer in state-societal relations in China?

**DOI:** 10.1186/s12992-018-0364-7

**Published:** 2018-05-16

**Authors:** Catherine Yuk-ping Lo

**Affiliations:** Southeast Asia Research Centre, College of Liberal Arts and Social Sciences, City University of Hong Kong, 83 Tat Chee Avenue, Kowloon Tong, Hong Kong

**Keywords:** HIV/AIDS securitization, Health NGOs in China, Global health initiatives (GHIs), Global Fund, United Nations Security Council (UNSC), Sustainable Development Goals (SDGs)

## Abstract

**Background:**

China has experienced unprecedented economic growth since the 1980s. Despite this impressive economic development, this growth exists side by side with the human immunodeficiency virus/acquired immune deficiency syndrome (HIV/AIDS) and severe acute respiratory syndrome (SARS) crises and the persisting deficiencies in public health provision in China. Acknowledging the prevailing health problems, the Chinese government has encouraged the development of health non-governmental organizations (NGOs) to respond to the health challenges and address the gaps in public health provision of the government. HIV/AIDS-focused NGOs have been perceived as the most outstanding civil society group developed in China. Considering the low priority of health policies since the economic reform, the limitation of the “third sector” activity permitted in authoritarian China, together with the political sensitivity of the HIV/AIDS problem in the country, this article aims to explain the proliferation of HIV/AIDS-focused NGOs in China with the usage of the securitization framework in the field of international relations (IR).

**Methods:**

The research that underpins this article is based on a desk-based literature review as well as in-depth field interviews with individuals working in HIV/AIDS-focused NGOs in China. Face-to-face interviews for this research were conducted between January and May in 2011, and between December 2016 and January 2017, in China. Discourse analysis was in particular employed in the study of the security-threat framing process (securitization) of HIV/AIDS in China.

**Results:**

This article argues that the proliferation of HIV/AIDS-related NGOs in China is largely attributed to the normative and technical effects of HIV/AIDS securitization ushered in by the United Nations Security Council (UNSC) and supported by the Global Fund to Fight AIDS, Tuberculosis, and Malaria (hereinafter Global Fund) observed in China. Despite depicting a positive scenario, the development of HIV/AIDS-focused NGOs in China generated by the international securitization efforts is largely limited. An internal and external factor was identified to verify the argument, namely (1) the reduction of international financial commitments, as well as (2) the fragmentation of HIV/AIDS-focused NGO community in China.

**Conclusions:**

This article shows that international securitization weakened with the rise of Chinese commitment on HIV/AIDS interventions. In other words, HIV/AIDS-related responses delivered by the national government are no longer checked by the global mechanism of HIV/AIDS; thus it is unclear whether these NGOs would remain of interest as partners for the government. The fragmentation of the HIV/AIDS community would further hinder the development, preventing from NGOs with the same interest forming alliances to call for changes in current political environment. Such restriction on the concerted efforts of HIV/AIDS-related NGOs in China would make achievement of the Sustainable Development Goals (SDGs) to foster stronger partnerships between the government and civil society difficult, which in turn hindering the realization of ending HIV/AIDS in the world by 2030.

## Background

China has experienced remarkable economic growth since the initiation of the economic reform in the 1980s. The average GDP growth in China was 9.6% annually during the first two decades of the economic reform [1]. The rising nation became the world’s leading manufacturing power in 2011, and it surpassed the United States as the world’s largest trading country [2]. Jakovljievic further predicted that the economic growth in China would continue at least up to the middle of the 21st century [3]. Despite such impressive economic performance, the provision of public health and the control of infectious diseases have been marginalized. Between the late 1980s and 2003, the Chinese national government public health spending as a proportion of total health spending plunged from 30% to just over 15% [4], despite the fact that China has outperformed other developing countries, such as other BRICS members, regarding the public health spending in nominal terms [5]. As argued by Yip and Mahal, Chinese political leaders in the above-mentioned period of time simply perceived health as “a consumption activity rather than a productive good and therefore was given lower priority in government funding” [4]. In this regard, the Chinese authorities deliberately curtailed the level of public health expenditures so that economic development could be pursued and the legitimacy of the one-party authoritarian regime bolstered.

Acknowledging the economic success in the past decades, HIV/AIDS nevertheless emerged as one of the “negative externalities” of public health threats brought about by the 1980s economic reform. HIV/AIDS was first discovered in China during the mid-1980s, the same period when the economic reform was just beginning. The epidemic was exacerbated in China in the 1990s mainly because of government-supported blood plasma selling, which did not follow adequate sterilization procedures. This lack of regulation resulted in the emergence of so-called AIDS villages (*Aizibing cun*) in Henan and its outlying provinces, where a large percentage of rural residents were infected with HIV/AIDS [6]. Having little benefit from the economic reform in ways that coastal provinces did during the 1980s, many residents in the central plains had little choice but to engage in the blood-plasma industry to improve their standard of living. Since 2000, HIV/AIDS has become a politically sensitive issue in China because of the international condemnation of the Henan blood scandal. According to the latest figures released by The Joint United Nations Program on HIV/AIDS (UNAIDS), 660,000 people were estimated to have HIV/AIDS in China in 2016 [7]. The country has the second largest HIV/AIDS population in Asia after India.

The Chinese government started addressing the HIV/AIDS problem after years of denial and underestimation of the HIV/AIDS prevalence in the country, especially among the most-at-risk populations (MARPs) including injecting drug users (IDUs), female sex workers (FSW), and men who have sex with men (MSM) [8]. However, national HIV/AIDS policies in the early years were very much driven in a top-down manner with government institutions (i.e. mainly the Chinese Centers for Disease Control and Prevention [China CDC hereinafter]) playing a leading role and with limited engagement of the “third sector” [9, 10]. Given the association of the disease with behaviors that are legally or socially unacceptable in the country (i.e., commercial sex work, injecting drug use, and men having sex with men), HIV/AIDS high-risk individuals refrain from using the related services provided by the government agencies because they wish to avoid being seized or humiliated by public security officials or by thugs hired by local officials [11]. In this regard, Gåsemyr claimed that the emergence of HIV/AIDS-focused non-governmental organizations (NGOs)^1^ in China during the early 1990s could fill the capacity gap of the government by implementing national HIV/AIDS policies and providing the related services for those MARPs that are hard to reach by the authorities [12]. Considering the low priority of health policies since the economic reform, the limitation of the “third sector” activity permitted in authoritarian China, together with the political sensitivity of the HIV/AIDS problem in the country, it is believed that the involvement of HIV/AIDS-related NGOs in China would be largely limited. This is however not the case. Over the last 15 years there has been a dramatic increase in the number of HIV/AIDS-focused NGOs operating in China. While Gåsemyr argued that the growth of HIV/AIDS-related NGOs is mainly attributed to the devastating health crisis of SARS in China and the new Chinese leadership in 2003 [12], the previous work cannot explain why a high degree of political recognition was observed in solely HIV/AIDS NGOs, instead of other health NGOs, such as TB or cancer NGOs in China. This article aims to fill this research gap with regard to the proliferation of HIV/AIDS-focused NGOs in China with the use of the securitization framework in the field of IR.

## Methods

### Research methodology: Discourse analysis

The research that underpins this article is based on desk-based literature review as well as in-depth field interviews with individuals working in HIV/AIDS-focused NGOs in China. Discourse analysis was in particular employed to study the securitization of HIV/AIDS in China. Potter argued that “discourse analysis has an analytic commitment to studying discourses as texts and talks in social practice…the focus is…on language as…the medium for interaction…One theme that is particularly emphasized here is the rhetorical or augmentative organization of talk and texts” [13]. Silverman pointed out that the intellectual ancestor of discourse analysis is John Langshaw Austin, the original inventor of the idea of speech acts that later became the core element in securitization theory [14]. Concerning discourse analysis and speech acts share the Austinian concern with the rhetorical structure of talk and texts, the founding scholars of securitization theory, Buzan, Wæver and Wilde explicitly stated, “the obvious method [to study securitization] is discourse analysis, since we are interested in when and how something is established by whom as a security threat” [15]. The authors also noted that the technique of employing discourse analysis to securitization studies is to “read[ing], looking for arguments that take the rhetorical and logical form defined here as security” [15]. The meaning of security is constructed when the political leaders (securitizing actors) frames an issue as an existential threat to the referent objects,^2^ claiming the issue as having an absolute priority on the government agenda, thereby invoking a right to handle such an issue with extraordinary/emergency measures to ensure the survival of the referent objects [16].

In this research, public speeches (in written form) related to HIV/AIDS problems, which were delivered by the top-level political leaders, were examined. A specific rhetorical structure had to be located in their discourses, consisting of either one or all of the following elements: (1) the existence of an existential threat; and/or (2) the perceived threat is challenging the survival of the states or people in the states (referent objects), and/or which it requires to (3) be urgently dealt with by new political measures of the respective country. An array of documents ranging from official documents, reports, newspaper articles was initially identified via the search engines of Google Scholar and ProQuest with the keywords of “HIV/AIDS”, “security threat”, and “China”. Nine key articles were consequently singled out to identify the textual discourses related to HIV/AIDS threat/security nexus in China, published or released between 1987 and 2011 (Table 1).

**Table 1:** Official documents, reports, newspaper articles for discourse analysis

Anderson D. Peking Daily Cautions against Western Threats of AIDS, Drugs. The Associated Press. (1987).	
Certain Regulations on the Monitoring and Control of AIDS (1988)	
China Has No Sources, Studies Show. Xinhua (1988)	
Prevention and Treatment of Infectious Diseases (1989)	
Speech by Executive Vice Minister of Health, Mr. Gao Qiang, at the HIV/AIDS High-Level Meeting of the UN General Assembly. Permanent Mission of the People’s Republic of China to the UN (2003)	
State Council Notice on Strengthening HIV/AIDS Prevention and Control (2004)	
Law on Communicable Disease Prevention and Control (2004)	
Regulation on AIDS Prevention and Control (2006)	
State Council Notice on Further Strengthening HIV/AIDS Prevention and Control (2010)	

### Primary data collection: Semi-structured interviews in China

A total of 47 semi-structured interviews were conducted along with the discourse analysis. Apart from the background information of the respective NGOs, the interview questions used in this study are categorized into three main dimensions: (1) the priority of HIV/AIDS on the government agenda; (2) the perceptions on the changing international and national financial commitment on HIV/AIDS interventions; and (3) civil society involvement and coordination in national HIV/AIDS policies. The first dimension aimed to ensure that the NGOs respondents were knowledgeable about the changing national government perceptions and policy priority towards HIV/AIDS in China, therefore, the researcher could rule out those comments given by unknowledgeable respondents so as to enhance the internal validity of the data collected. The second dimension was included to study their perceptions on the level of HIV/AIDS funding and effect (if any) on the service provision. The third dimension aimed to discover the actual level of involvement of NGOs in national HIV/AIDS interventions as well as the level of coordination and cooperation between different NGOs working on HIV/AIDS.

Two types of non-probability sampling—purposive sampling, including “theoretical sampling” and snowball sampling—were employed in the selection process of key informants. For purposive sampling method, samples were selected based on the “logic” or “commonsense” of the researcher in terms of the target population, its elements, and the purpose of the study. In this study, the target population consisted of the individuals working in HIV/AIDS-related NGOs at global, national, and grassroots levels. In the course of the data collection, “theoretical sampling” was also applied to understand the subjects from a theoretical perspective. Hence, academic scholars who have conducted research on HIV/AIDS policies in China were also included in the interviewee list. Interviewees were also recruited via snowball sampling. The researcher collected data on the few members, and each respondent was asked to suggest people whom the respondent believed to be the most influential for interviewing.

Interviews for this research were undertaken between January and May in 2011, and between December 2016 and January 2017 with leaders or senior officers working on HIV/AIDS in China, including 15 international NGOs, nine self-help groups, four GONGOs, five advocacy groups, two national NGO, one independent consultant, two academia, and two networks (See Table 2). Twenty-six respondents were pinpointed based on the list of the *2009/2010 China HIV/AIDS NGO Directory* (*Zhongguo aizhibing shehuizuzhi minglu*) published by a quasi-NGO, China HIV/AIDS Information Network, operated in Beijing. The rest of the respondents in this research were recruited via the technique of snowball sampling and theoretical sampling. There were some exceptional cases regarding the inclusion of some interviewees in this study. Amongst the NGO respondents, three of them were based in Hong Kong. These individuals were interviewed because of their direct participation in the HIV/AIDS interventions in the Chinese cities in the past years, as well as their close relationship with the government officials and other HIV/AIDS-related NGO members in Mainland China. In addition, four government officials working in the National Health and Family Planning Commission (NHFPC) were interviewed, including the National Center for AIDS/STD Control and Prevention (NCAIDS), China CDC in Beijing, Shanghai, and also Zhejiang. The inclusion of the government officials was made to have a better understanding of the stance of the national government towards HIV/AIDS issues. Ethical approval for this research (including subject participation) was provided by the City University of Hong Kong Human Subjects Ethics Committee, under the project *Asian Diseases in International Affairs*, Ref: 2A-54-201,303 (H00112).

**Table 2:** Categorization of respondents in China

Number of interviewees	Types of organizations	Locations
15	INGO	Beijing, Kunming
9	Self-help group	Beijing, Shanghai, Kunming
4	GONGO	Beijing
5	Advocacy group	Beijing, Shanghai, Kunming
2	National NGO	Beijing
1	Independent consultant	Kunming
2	University researcher	Beijing, Hong Kong
2	Network	Beijing, Kunming
3	Hong Kong-based NGO	Hong Kong
4	CCDC/NHFPC	Beijing, Shanghai, Zhejiang

## Results

### Literature review: State-societal relations and HIV/AIDS-related NGOs in contemporary China

Civil society (*Minjian shehui*)^3^ was not considered as part of the political system in the pre-modern era; the emergence of a relatively independent civil society is a product of modern China. The changing socio-economic relations brought about by “Open Door Policy” resulted in the recognition and growth of civil society for the first time in the Chinese history [17]. Despite the early emergence of civil society, the 1999 *Falung Gong* incident showcased the destructive power of civil society in the eyes of the Chinese government, worrying a robust, well-organized, and out-of-control civil society could overthrow and replace the CCP ruling position. Meanwhile, the authorities realized the supplementary role NGOs could perform in policy implementation. The Chinese government has thus resolved such dilemma by adopting a “state-led” approach to manage civil society in China. Based on Frolic’s concept of “state-led civil society”, civil society is created by and belong to the state, thus the independence and autonomy of civil society are at all time bounded by the state [18]. The state has the role of legitimating social organizations, demanding a disciplined partnership. Accordingly, antagonism inherited in the Western concept of civil society is not allowed to exist in the state-society relations in Chinese authoritarian regime; any alternative force to the state is considered as an attempt to curtail or overturn its political legitimacy and power.

Accordingly, the state apparatus has managed the numbers and restricted the growth of both international and grassroots NGOs via various legislative means, thereby allowing NGOs to operate openly but at the same time keep the organization growth in check [19, 20]. International non-governmental organizations (INGOs) in China are regulated by the 1989 Interim Procedures on Foreign Chambers of Commerce (*Waiguo shanghui guanli zhanhang guiding*) [21]. This law stipulates that INGOs can only set up one branch office in China, restricting the geographic scope (and thus political or social influences) of INGOs operating in the country. In regulating the rapid influx of foreign endowment in China in the early 2000s, especially in the realm of HIV/AIDS interventions, the State Council promulgated the Regulations on the Management of Foundations (*Jijin guanli tiaoli*) in 2004.^4^ Despite these regulations, the Chinese authorities had relatively given more autonomy to INGOs than grassroots NGOs operating in its territory, since the government could access international expertise and tap foreign money to tackle with the emerging social problems in China [22]. Such degree of autonomy is nevertheless problematic after the promulgation of Foreign NGOs Management Law (*Jingwai fei zhengfu zuzhi guanli fa*) in April 2016. The 2016 law stipulates that INGOs operating in China must register with public security officials, and must not engage in political or religious activities that damage “China’s national interests” or “ethnic unity”. International community and western governments perceived the new law as a reinforcement of restriction of the numbers and scopes of foreign entities operating in the authoritarian regime.

Grassroots NGOs are regulated by the Regulation on Registration and Administration of Social Organizations (*Shehui tuanti dengji guanli tiaoli*). According to the 1998 regulation (amended in February 2016), a NGO must possess a minimum asset of 100,000 yuan and have a “professional management unit” (*Zhuguan danwei*) that acts as a supervisory body to the organization in order to register under the Ministry of Civic Affairs (MOCA).^5^ Fulfilling these two requirements is very formidable for grassroots NGOs. The financial situation is problematic in many grassroots NGOs because of the limited source of funding. On the one hand, most grassroots NGOs receive limited financial support from the government, as they usually do not have political ties with the government. Grassroots NGOs also seldom receive donations from local communities because (1) newly developed NGOs do not have good track records that enable them to win the trust of the locals, and (2) donors cannot receive tax breaks for their donation to unregistered NGOs [23, 24]. In addition, most government departments simply reject their applications due to fear of consequences of taking responsibilities for grassroots NGOs. Many grassroots NGOs are hence often unregistered or registered as business entities with the Ministry of Industry and Commerce (MOIC) [25].^6^ In addition to the abovementioned restrictions, the Regulation also bans “similar organizations” coexisting at the various administrative levels [21], facilitating the management and even control the legal status of grassroots NGOs in China.^7^ Having official registration, to certain extent, is sine qua non to the survival of organizations as the legal status entitles NGOs as official recognized entities to receive legal and financial supports from the government; unregistered NGOs are officially perceived as illegal that would be subjected to prosecution and coercion by the state apparatus.^8^

Considering a restrictive engagement of grassroots NGOs in China, instead of grassroots NGOs, early HIV/AIDS-related responses at societal level were largely conducted by GONGOs [26],^9^ such as The Chinese Association of STD and AIDS Prevention as well as the Chinese Preventive Medicine Association. A few NGOs working on HIV/AIDS emerged with the upsurge in the number of infections in the country during the 1990s. Established in 1994 by Dr. Wan Yanhai, the Beijing-based AIDS Action (*Aizhixing*) was one of the earliest and prominent HIV/AIDS-related NGOs operating in China.

A dramatic increase in the number of HIV/AIDS-focused NGOs has nevertheless been observed in China since 2003. According to the figures released in the *2009/2010 China HIV/AIDS NGO Directory* (*Zhongguo aizhibing shehuizuzhi minglu*), the number of registered and unregistered NGOs working on HIV/AIDS issues in China increased from 52 before 2003 to over 600 in 2010 [27]. Considering the low priority of health policies since the economic reform, the limitation of the “third sector” activity permitted in authoritarian China, together with the political sensitivity of the HIV/AIDS problem in the country, it is intriguing to uncover the reasons for the growth of HIV/AIDS-focused NGOs in China since 2003.

Gåsemyr argued that the growth of HIV/AIDS-related NGOs is mainly attributed to the devastating health crisis of SARS in China and the new Chinese leadership in 2003 [12]. Having said that the Chinese government realized the impacts health problems or infectious diseases could have on its economic and social development, the previous work cannot explain why a high degree of political recognition was observed solely in HIV/AIDS NGOs, instead of other health NGOs, such as TB or cancer NGOs in China. Considering the regime type of the government, allowing the growth of local NGOs would in turn potentially attenuate the supremacy of the Chinese Communist Party (CCP) in ruling the country or erode the autonomy of the state, why the Chinese government tolerated, allowed, or even encouraged the growth of NGOs in fixing HIV/AIDS problems, especially the epidemic has been viewed as a politically sensitive issue in China? How can one account for and understand the phenomenon?

Answering the key question with an IR perspective, this article argues that the rapid development of HIV/AIDS-related NGOs in China is attributed to the effects of HIV/AIDS securitization at the international level since 2000. Considering one of the most influential theories developed in the field of IR, securitization describes the process of defining an issue as a security threat (speech acts) and the corresponding political responses to block the adverse development of the perceived threat (emergency measures). This article particularly pinpoints the UNSC and the Global Fund, which provide normative and technical supports, to study this endeavor. Normative influences in this article refer to the effects of security-threat discourses, primarily Resolution 1308 of the UNSC, on the national government’s perception on HIV/AIDS problems, whereas technical influences refer to the effects of global emergency measures, in particular the Global Fund, on the funding and management of HIV/AIDS-related policies at the state level [28]. This article aims to demonstrate the ways in which international HIV/AIDS securitization has served as a game changer in altering state-society relations in China. Drawing on literature reviews and fieldwork materials, this article further argues that the proliferation of HIV/AIDS-focused NGOs in China generated by the international securitization efforts is nevertheless largely limited owing to the reduction of international financial commitments. The fragmentation of HIV/AIDS-focused NGO community is another factor hindering the development of a full-fledged third sector in the country.

### Literature review: HIV/AIDS securitization in the international level

Securitization in the IR discipline is a model outlining the process of framing an issue as a security threat and the corresponding political reactions to block the adverse development of the perceived threat. Employing the theory, one is required to look for three criteria to determine whether an issue is a threat to the country and its people, including 1) discourses uttered by top-level political leader (securitizing actor) declaring a particular referent objects as an existential threat to security; 2) acceptance by the targeted audience convinced of its potential to be an existential threat (audience acceptance); and also 3) redirections of existing financial resources, new policies, or practices (emergency measures) are implemented to address the issue following the security-threat claim [15]. In other words, the resolution of the “existential threat” posed by “X” takes precedence over other problems [29].

Numbers of security studies scholars believed that HIV/AIDS is the first infectious disease being securitized by the international institutions and national governments as the abovementioned criteria were all fulfilled in the case of HIV/AIDS [9, 30–32]. Speech acts concerning HIV/AIDS as a security threat were first identified in a UNSC meeting in January 2000.^10^ In July of the same year, the UNSC passed Resolution 1308, acknowledging the urgent need to address HIV/AIDS, which threatens the stability and security if left unchecked [33]. It is perceived that the particular security framing of HIV/AIDS gained acceptance by the national governments (audience of the HIV/AIDS security-threat claim) since 189 countries unanimously adopted and signed the Declaration of Commitment on HIV/AIDS in 2001. The urgency of addressing HIV/AIDS problems was reinforced in the special United Nations General Assembly Special Session (UNGASS) devoted to HIV/AIDS that has taken place every 5 years since 2001 [34–37]. The abovementioned events are significant because they represented the first time that the UNSC General Assembly devoted an entire session to a single disease, and the first time that the international political authorities framed HIV/AIDS as a global health threat to national and international security instead of solely as a developmental or public health problem [38].^11^

The 2000 rhetorical act was backed by operational emergency responses in terms of the augmentation of the global HIV/AIDS spending since 2000. The amount was exponentially surged from about US$900 million in 1999 to US$16 billion by 2009; and to an estimated US$19 billion in 2013 [39]. Several HIV/AIDS-related funding agencies and health programs formulated after 2000, also referred to Global Health Initiatives (GHIs), brought in additional monetary resources for HIV/AIDS [40].^12^ Perceiving as the most prominent GHIs, the Global Fund pledges to accelerate the end of the three epidemics, [HIV/]AIDS, tuberculosis, and malaria by providing financial support (US$4 billion annually) for low-income countries to respond to the three diseases [41]. Undoubtedly, the introduction of the abovementioned GHIs has brought about unprecedented levels of funding for diseases, in particular HIV/AIDS, in countries that have insufficient resources, whether because of poor socio-economic development or lack of political will, to orchestrate appropriate responses to the intractable disease.

Acknowledging international securitizing efforts on HIV/AIDS, this article illustrates the normative effects of Resolution 1308 of the UNSC and the technical influences of the Global Fund on national-level HIV/AIDS responses. China is selected as a case study to investigate this endeavor. China’s response to the HIV/AIDS problem changed from an ignorance of “global evidence-based policy recommendations and international advice” into “one of our success stories [in global HIV/AIDS fight] during this past 15 years,” as claimed by Michel Sidibe, executive director of the UNAIDS [42, 43]. The subsequent sections demonstrate the ways in which the securitization effort in the international level acted as a game changer for HIV/AIDS-focused NGOs in participating in national HIV/AIDS undertakings in China.

### Chinese case study

#### Discourse analysis: Normative influences and the role of the UNSC

In China, many of the first cases of HIV/AIDS since 1985 occurred among foreigners and homosexuals. Therefore, the official discourses at that time promoted the idea that HIV/AIDS only affected non-local Chinese people and foreigners, asserting “drug taking, alcoholism, robbery, homicide, suicide, divorce, prostitution, homosexuality, syphilis, AIDS…These kinds of Western social ills come from their ideology”, and also claiming “China had no sources of HIV/AIDS” [44, 45]. The rhetorical acknowledgement of HIV/AIDS as a health security threat to the Chinese population was largely absent during the early period of time. Ramiah also pinpointed that the Chinese leadership was extremely reluctant to acknowledge HIV/AIDS as a problem and securitize HIV/AIDS in China prior to the 2000s [46].

One probable reason for the resistance to HIV/AIDS securitization is economic: the period of early HIV/AIDS outbreak coincided with the initial years of Open Door Policy and economic reform (*Gaige kaifang*) of China to the outside world. The thirst for economic growth and performance preceded the urgency of disease management: local authorities feared their respected jurisdictions would lose external or foreign investment if the full extent of the problem were known, or that they would be punished by superiors for failing to prevent the HIV/AIDS spread. The “cover-up” practice also occurred during the early outbreak of SARS in China in 2003, for the infectious disease clearly posed a negative impact on the Chinese flourishing trade and tourist industry.

In light of the emerging HIV/AIDS epidemic in China, the 2000 international HIV/AIDS securitization has been well accepted by the Chinese government. China was one of the 189 signatories to the 2001 Declaration of Commitment on HIV/AIDS, showing its commitment to follow suit the global consensus framework to response to the HIV/AIDS threats. More importantly, the 2001 Declaration included a set of goals on which the national governments agreed to achieve within a certain timeframe (i.e. most targets should be fulfilled either by 2003 or by 2005).

In response to the designated targets, the security-threat framing of HIV/AIDS has been fully adopted in the official discourses of Chinese leaders and in official documents since 2003. Previously viewing HIV/AIDS as a “Western disease” or “non-Chinese disease”, Executive Vice Minister of Health Gao Qiang stated in the HIV/AIDS High-Level Meeting of the UN General Assembly that “HIV/AIDS is a *common enemy of the whole mankind *as it seriously *threatens public health and safety*. The Chinese government has *attached great importance to HIV/AIDS* prevention and treatment and has treated it as a* strategic issue for social stability, economic development, national prosperity and security*, making it a *first priority* of the government work” [emphasis added] [47]. The Chinese President Hu Jintao once claimed in a 2003 public speech that “HIV/AIDS prevention, care and treatment is a *major issue pertinent to the quality and prosperity *of the Chinese nation”; whereas Premier Wen likewise asserted that “dealing with HIV/AIDS as an *urgent and major issue* is related to the *fundamental interests of the whole Chinese nation*” [emphasis added] [47]. The ideas in the speeches were restated in the official document entitled State Council Notice on Strengthening HIV/AIDS Prevention and Control (*Guowuyuan guanyu qieshi jiangqiang aizibing fangzhi gongzuo de tongzi*). This 2004 document explicitly demonstrated the determination of the Chinese authorities in grappling with the disease and stated that “HIV/AIDS prevention and control is linked to *economic development, social stability, and national security and prosperity*. Long-term commitment to respond to HIV/AIDS is hence necessary” [emphasis added] [48]. China’s HIV/AIDS prevention and control policies were further strengthened in the State Council Notice on Further Strengthening HIV/AIDS Prevention and Control (*Guowuyuan guanyu jinyibu jiangqiang aizibing fangzhi gongzuo de tongzi*) in late 2010, claiming that “the prevention and control of HIV/AIDS is related to the people’s physical health and social and economic development, as well as to *national security and the rise and fall of the nation.* The Party Central Committee and the State Council have *always attached great importance* to the prevention and treatment of HIV/AIDS” [emphasis added] [49]. Based on the discourse analysis of the official documents and newspaper articles, it is argued that Chinese national leaders followed suit the international move (i.e. UNSC Resolution 1308) to securitize HIV/AIDS in the country, framing HIV/AIDS as a threat with social, political, economic, and security implications.

Such an alteration in perception toward the epidemic brought about the political recognition of the HIV/AIDS-focused NGOs, which has also been highlighted by multiple statements delivered by various top-level government officials since 2003. In 2004, Vice Premier Wu Yi stated, “We should mobilize all the partners in society to participate in the fight against HIV/AIDS. We need to improve our policies and strategies to build a better environment for the society to participate in the response, and try our best to facilitate the involvement of all sectors” [49]. In 2011, Vice Minister of Health Yin Li publicly proclaimed “civil societies play an indispensable role in effective HIV/AIDS interventions led by the government” [50]. Premier Li Keqiang even promoted tax break for NGOs specializing in HIV/AIDS prevention, claiming the role of NGOs in HIV/AIDS prevention “is an irreplaceable and unique force” [51]. To a certain extent, these aforementioned statements demonstrate the state’s recognition of NGOs’ works and its willingness to engage with NGOs in national HIV/AIDS interventions, although the political space of involvement is by and large bounded by the state [52].

The previous section illustrates the change in the perception of the Chinese government toward the HIV/AIDS problems: from denial to the acceptance of the threats that could be posed by the disease on the country and its people. Such threat recognition also brought about the acknowledgement that the government alone could not resolve the HIV/AIDS problems because of the complicated nature of the epidemic; the involvement of civil society groups was a globally recognized recommendation leading to the successful containment of the epidemic. Nevertheless, verbal recognition is not sufficient to enhance the engagement and development of HIV/AIDS-focused NGOs, especially in the preexisting authoritarian government structure; the governance and funding mechanism for HIV/AIDS responses requires alternation in China. As illustrated in the following section, this was the mechanism of the Global Fund that enhanced the funding for HIV/AIDS-focused NGOs, facilitating the involvement of civil society groups in translating the rhetorical acts into emergency responses of HIV/AIDS in China.

#### Key informant data: Technical influences and the role of Global Fund

Among the international funding agencies emerging after the HIV/AIDS securitization launched by the UNSC, the Global Fund was the most renowned major contributor to the HIV/AIDS interventions in China. The Global Fund finally accepted the first proposal submitted by the Chinese government on HIV/AIDS interventions in late 2003, after the previous proposals being turned down twice in 2002 [52]. Between 2003 and 2012, the Chinese government has received over US$8 billion in grants from the Global Fund; approximately US$324 million were disbursed to HIV/AIDS intervention programs [41]. Further information regarding the Global Fund on HIV/AIDS in China is illustrated in Table 3.

**Table 3:** Global Fund Grant Portfolio for HIV/AIDS in China [34]

Round	Grant title	Requested amount (in USD)	Lifespan
3	China CARES (China Comprehensive Aids Response): A Community- Based HIV Treatment, Care and Prevention Program in Central China	97,888,170	2004–2009
4	Reducing HIV transmission among and from vulnerable groups and alleviating its impact in seven provinces in China	63,742,277	2005–2010
5	Preventing a new wave of HIV Infections in China	28,902,073	2006–2011
6	Mobilizing Civil Society to Scale Up HIV/AIDS Control Efforts in China	14,395,715	2007–2012
8	Reaching vulnerable migrants with HIV/AIDS prevention and care services in seven provinces in China	61,413,199	2009–2014
RCC^a^	China Global Fund AIDS Program	509,000,000	2010–2015

^a^RCC stands for Rolling Continuation Channel Program

Intervention of the Global Fund’s funding mechanism was deemed to transform the primary HIV/AIDS governance in China [53]. The Global Fund required the governments to set up a country-level Country Coordination Mechanism (CCM) to construct the grant proposals in line with the national HIV/AIDS-related strategies, manage the use and distribution of grants, and also oversee the implementation of successful applications. As per the requirement of the Global Fund, CCM should consist of a broad representation from the government, NGOs, multilateral and bilateral agencies, and private sector [54]. In the existing government structure in China, grassroots NGOs have limited channels to participate in policymaking and implementation processes. Thus, the multi-sectoral structure of CCM contributed to enhancing the government’s commitment to involve NGOs in national HIV/AIDS prevention and control measures [55]. Two among the 22 seats in Chinese CCM were reserved for the representatives of HIV/AIDS-related grassroots NGOs and people living with HIV/AIDS are a case in point [56]. Considering the existing government structure does not allow the full involvement of NGOs in China, the CCM provided valuable opportunities for civil society groups to engage in HIV/AIDS-related policymaking.

The involvement of NGOs in national HIV/AIDS programs was notably highlighted in Round 6 of the Global Fund in China, since the theme of the grant proposal submitted by the CCM was directly linked to the development of civil society groups (Refer to Table 3). In the course of the interview, a director of a Hong Kong-based NGO commented on the profound influence of the Round 6 on the NGO development: “Round 6 of the Global Fund acts as a pushing force for the government to involve more grassroots NGOs in HIV/AIDS interventions” (national NGO-1). In line with the respondent’s opinion, Kaufman argued the Round 6 in particular “was seen by many as a further mechanism to institutionalize HIV/[AIDS] NGOs roles in China’s [HIV]/AIDS response [42].”

In addition to the increase in participation, financial support for NGOs working on HIV/AIDS-related programs surged through the Global Fund. As the Principal Recipient (PR) of the country, the Chinese government (i.e., China CDC or the NHFPC) was required to disburse a designated portion of the grants to HIV/AIDS-related NGOs (in the form of sub-recipients) operating in the country [41]. Following the instruction, 20% of the total grant was allocated to support NGOs in Round 3 and 4, whereas 50% of its grant was distributed to NGOs in Round 5 of the Global Fund [56]. In line with the theme of Round 6 entitled “Mobilizing Civil Society to Scale Up HIV/AIDS Control Efforts in China” proposed by the Chinese CCM, the proportion granted to HV/AIDS-related NGOs reached 100% [52]. Owing to the constructive political atmosphere and promising financial resources in the country, a burgeoning number of individual-organized HIV/AIDS-focused NGOs was established in China; over 43% of the number of grassroots NGOs increased in China between 2003 and 2004 [57].

The suspension of the Global Fund in 2011 demonstrated that the funding mechanism to some extent held the Chinese government accountable for the financial supports of HIV/AIDS-focused NGOs. The Global Fund once suspended its funding to Chinese HIV/AIDS programs in early 2011 because the Chinese CCM failed to allocate 35% of the US$283 million HIV/AIDS grant to grassroots NGOs as pledged; less than 11% was distributed to HIV/AIDS-focused NGOs [58]. A program officer of a NGO mentioned the failure of CCM to allocate the right portion of the Global Fund grant to grassroots NGOs: “Government officials and representatives from GONGOs dominate the CCM. Only a few representatives are from grassroots NGOs and civil society” (advocacy group-1). The improper management and use of the Global Fund was even admitted by a senior government official in the Shanghai CDC in times of the interview:

*First, the Global Fund required at least 25 percent to 30 percent of the grant should be given to grassroots NGOs. However, the government failed to achieve this requirement. Second, the management fee in some provinces was too high. Third, one of the elements in the RCC program was related to budget aggregation (i.e., monetary contribution by both the national government and the Global Fund). However, the Chinese government did not allocate enough money for the aggregated budget* (CCDC/NHFPC-1)*.*

The determination of the Chinese government to resolve the suspension of the Global Fund was observed in the course of fieldwork research. During a face-to-face interview inside the office building of the Ministry of Health (renamed National Health and Family Planning Commission in 2013) in May 2011, the respondent told the investigator “the representatives of the Chinese government and the Global Fund representatives are meeting upstairs to negotiate for the resumption of the 2011 grants” (CCDC/NHFPC-2). The dispute was eventually resolved; the Chinese government promised to allocate at least 25% of the Global Fund grant to NGO work [59]. In this regard, the Global Fund mechanism contributed to the political and financial supports of NGOs, thereby resulting in the proliferation of HIV/AIDS-specific grassroots NGOs in China, and also the proactive involvement of NGOs in national HIV/AIDS undertakings. In other words, international securitization altered the HIV/AIDS policy in China, allowing numerous grassroots-level NGOs working on HIV/AIDS to grow in a prevailing political system with “bounded autonomy”. An injecting drug use self-help group leader believed the upsurge in the involvement of civil society also resulted in the improvement of state-societal relations, stating, “In the past, the relationship with the government was not good, but it becomes much better now. The government sent officials to visit our center to learn the management strategy of our organization” (self-help group-1). Another NGO interviewee in Beijing also claimed that “an increase in the acceptance of the government, especially the Ministry of Health, on grassroots NGOs, [is observed in China]. The Ministry of Health does open the opportunity for NGOs to cooperate with the government” (advocacy group-2). Having said that the improvement of state-societal relations, it is noted that the Chinese state is not a monolithic actor, but a conglomeration of departments and officials with competing and sometimes conflicting agendas in the national and sub-national levels [22]. A Beijing NGO leader pinpointed this phenomenon, stating, “The Ministry of Health is fine to cooperate with those NGOs that can help, regardless of whom. However, state security bureau usually holds a relatively hostile view on the existence of grassroots NGOs since the bureau is concerned about social stability” (advocacy group-2).

Considering HIV/AIDS as a health threat to the country and its people, the recognition and involvement by the central government and the NHFPC and other related government agencies is of particular vital to the proliferation of HIV/AIDS-focused NGOs in a state-dominated society in the current political regime. Despite depicting a positive scenario, this article further illustrates that the development of HIV/AIDS-focused NGOs in China generated by the international securitization efforts is largely limited. In other words, the growth of the number of NGOs working on HIV/AIDS in China would be ceased or even decreased in long term, and also the political space for grassroots to involve in HIV/AIDS policy making and implementation would be further restricted. An internal and external factor was identified to verify the argument, namely (1) the reduction of international financial commitment, as well as (2) the fragmentation of HIV/AIDS-focused NGO community in China.

## Discussions

### Obstacles of the development of HIV/AIDS-related NGOs in China

#### External factor: The reduction of international financial commitment

China resembles some developing countries in that it has faced the problem of decreasing international HIV/AIDS financial commitments in supporting its national HIV/AIDS responses [60]. Given the 2008 Global Financial Crisis and the continuous economic boom in China, international and bilateral funding agencies argue that China should be the donor instead of the recipient of international HIV/AIDS-specific grants and loans [52]. In the course of interview, respondents in Ford Foundation, Médecins Sans Frontières (MSF), and Clinton Foundation in China mentioned that the INGOs reduced the financial support on HIV/AIDS programs or even withdrew from China, whereas UK Department of International Development (DFID), Oxfam, and Bill & Melinda Gates Foundation, have retreated from China since 2013 (INGO-1). The financial support for HIV/AIDS-related NGOs in China is further shrunk because of the retreat of the Global Fund in 2014. A HIV/AIDS specialist working in an INGO and a program manager of a national NGO recalled the profound impact of the retreat of Global Fund on HIV/AIDS-related NGOs:

*The Global Fund stopped the phase two of the consolidated program suddenly (With a total of approximately 500 million, only half of the amount was implemented in China in the end). As a result, the number of NGOs working on HIV/AIDS decreased drastically from 1,000 to 500 between 2013 and 2014* (INGO-1)*.*

*Owing to a lack of international and national HIV/AIDS funding, the number of NGOs working on HIV/AIDS was drastically reduced; many NGOs and CBOs were simply closed down, only a few of them could barely survive with the support by other funding sources* (national NGO-2).

The retreat of the Global Fund has subsequently created a policy vacuum for the Chinese government in taking the lead in managing the development of HIV/AIDS-related NGOs in China. Mimicking the funding mechanism of the Global Fund, the State Council launched the China AIDS Fund for Non-Governmental Organizations (CAFNGO) (*shehui zuzhi canyu aizibing fangzhi jijin*), providing financial resources for HIV/AIDS-related NGOs via the submission of grant proposal starting from 2017 [61]. The reduction of the Chinese government’s reliance on the Global Fund implies that the HIV/AIDS-related responses delivered by the national government are no longer checked by the external mechanism. The authoritarian regime is probably the only agency defining/restricting the political space that NGOs could work in. Explaining the interaction between the Chinese authorities and NGOs, a university professor in Beijing suggested: “In normal circumstances, local CDC staff hand over treatment delivery work to grassroots NGOs. Nonetheless, CDC officials intend to keep these NGOs small, giving them just enough money to run the programs, so that they will not grow too strong to pose potential threats to the government” (academic-1). Along with the weakening of international securitization efforts and the rise of Chinese government’s involvement in managing NGOs in the post-Global Fund era, the continuous proliferation of NGOs is further complicated by the fragmented nature of HIV/AIDS-focused civil society groups in China.

#### Internal factor: Fragmented HIV/AIDS-focused NGO community in China

It is argued that the existing NGO registration system, as previously mentioned, has brought about a fragmented NGO community in China. Apparently, government-organized non-government organization (GONGO) and registered NGOs working on HIV/AIDS are included in HIV/AIDS national policy implementation processes, whereas excluding unregistered grassroots NGOs that are perceived as illegal or “anti-government” organizations. Based on the nature of services that individual HIV/AIDS-focused NGOs provided, service providers are preferred by the state, whereas NGOs serving as advocates of human rights and agencies providing legal services for HIV/AIDS-infected people would be subjected to prosecution and coercion. A HIV/AIDS specialist working in an international NGO in Beijing stated, “During a 2011 HIV/AIDS NGO meeting, the Chinese government clearly claimed that the authorities would like to work with NGOs/CBOs conducting service delivery, but not with those working on HIV/AIDS-related human rights or gender issues” (INGO-1). To further illustrate this point, the Chinese political leaders publicly praised a Guangxi-based NGO named AIDS Care China, recognizing the work the organization had been done in HIV/AIDS prevention, meanwhile stifling numbers of HIV/AIDS activists through imprisonment, house arrest, or assault. Dr. Wang Yanhai and Dr. Gao Yaojie were two of the prominent Chinese HIV/AIDS activists that fled to the United States in 2009 and 2010, respectively, due to the recurrent pressure and disturbance exerted by Chinese authorities owing to their advocacy work. In June 2015, a HIV/AIDS-related legal aid NGO, Beijing *Yirenping*, was raided, and two of its activists were detained. In this regard, the continuous inclusion/exclusion selection process would weaken unregistered or advocacy groups that conceived as threats to the legitimacy of the authoritarian regime [62], while preserving NGOs that are willing to under control by the state apparatus and prepared to accept the regime’s policy measures. For the sake of reaching a *modus vivendi*, these NGOs usually display self-limiting behaviors, avoiding actions that would be interpreted as threats to the regime [20], such as having close relationship or cooperation with NGOs being “excluded” by the state apparatus.

While a formal local network for HIV/AIDS-related NGOs or civil society groups has been established in many countries in Asia, such coordination mechanism between non-state actors is largely absent in China.^13^ The fragmentation of HIV/AIDS NGO community is likewise observed in the course of interview sessions. Amongst the interviewees, the majority of the NGO respondents believed that cooperation dramatically increased between the government and NGOs working on HIV/AIDS, especially in the areas of treatment, care, and prevention. Only a few of them nevertheless stated that communication and coordination occurred amongst NGOs in policy implementation. A national-wide NGO respondent claimed that “We seldom cooperate with other NGOs or INGOs; we have our own teams to do the work” (national NGO-3). Some grassroots NGO respondents in Shanghai and Kunming expressed their unwillingness to cooperate with so-called “unauthentic” NGOs or NGOs having so-called “government background”:

*Few NGOs have cooperative relationship. There are lots of “fake” NGOs in China. The CDC found some patients to set up grassroots NGOs and then through the name of such NGO to apply for the Global Fund. After they received the money, CDC would become the one who controlled the funding* (self-help group-2).

*Eighty-five percent of the HIV/AIDS-focused NGOs in Yunnan have government background…for those NGOs that are funded by the Global Fund or China-Bill Gates Projects, these are organizations with the government background…or if you read their introduction saying “with the help and support of government”, you will know these are NGOs with the government background* (self-help group-3).

A “we-they” distinction has apparently prevailed amongst the HIV/AIDS NGOs community in China, reinforcing the atomization of HIV/AIDS-focused NGOs, discouraging the proliferation of civil society in China. Horizontal relationship amongst NGOs working on HIV/AIDS is relatively weak and ill-organized, thereby avoiding these organizations joining hand to call for favorable political environment or monetary supports to continue their operation in China. Owing to the reduction of international financial commitments, as well as the fragmentation of HIV/AIDS-focused NGO community in China, the proliferation of HIV/AIDS-focused NGOs in China generated by the international securitization efforts is nevertheless largely limited in long run. Such restriction on the concerted efforts of HIV/AIDS-related NGOs will inevitably pose challenges to China to fulfill the pledge of the 2030 Agenda to end HIV/AIDS as one of the targets of the Sustainable Development Goals (SDGs) [63].

## Conclusion

HIV/AIDS was officially announced as a security issue in 2000 by the UNSC Resolution 1308, which claimed that the pandemic could pose security threats if left unchecked. The rhetorical security-threat claim resulted in the burgeoning number of GHIs organized by the international and bilateral organizations operating at the state level. This article illustrates the ways in which the UNSC Resolution 1308 and the Global Fund influencing the HIV/AIDS governance in China. Rhetorically acknowledged the HIV/AIDS problems and the irreplaceable role of HIV/AIDS-focused NGOs in combating the disease by the Chinese government, the monetary and technical supports by the Global Fund enabled HIV/AIDS-focused NGOs to participate in the national policymaking process, facilitating the proliferation and development of civil society in China.

This success nevertheless does come with concerns regarding sustainability. This article shows that international securitization weakened with the rise of Chinese commitment on HIV/AIDS interventions. In other words, HIV/AIDS-related responses delivered by the national government are no longer checked by the global mechanism of HIV/AIDS; thus it is unclear whether these NGOs would remain of interest as partners for the government [64]. The fragmentation of the HIV/AIDS community would further hinder the development, preventing from NGOs with the same interest forming alliances to call for changes in current political environment. Such restriction on the concerted efforts of HIV/AIDS-related NGOs in China would make achievement of the SDGs to foster stronger partnerships between the government and civil society (goal 17) difficult, which in turn hindering the realization of ending HIV/AIDS in the world by 2030 (goal 3).

This research has several limitations. Firstly, there is a lack of available information regarding the operation of local or grassroots HIV/AIDS-specific NGOs that are in particular helping MARPs in China, owing to the political sensitivity of HIV/AIDS, the unregistered status of NGOs working on HIV/AIDS, the increasing restrictive environment for NGOs operating in China especially under the Xi administration, together with the social stigma attached to their service objects as well as the NGOs themselves as the service providers. Secondly, it is difficult to conduct longitudinal study on the development of HIV/AIDS-specific NGOs in China. The researcher realized that many of the NGOs that had been interviewed in 2011 were no longer under operation in the follow-up fieldwork in 2017, either because these NGOs could not secure sustainable funding, or they were cracked down by local authorities for advocating HIV/AIDS-related human rights or gender equality, or having very close links to foreign (western) organizations. Further research therefore needs to be conducted to remedy these technical weaknesses. It is suggested that comparative research could be conducted on the effects (if any) of the regime types, such as China and Thailand, and the level of socio-economic development of the country, such as China and Singapore, on the effects of international HIV/AIDS securitization in the national levels.

## Footnotes

1 Here NGOs does not include government-organized non-governmental organizations (GONGOs).

2 Referent objects refer to “things that are seen to be existentially threatened and that have a legitimate claim to survival.” See Buzan B et al., Security: A New Framework for Analysis, p. 36.

3 “Civil society” is often translated as “*Minjian shehui*”, “*Shiming Shehui*” and “*Gongming Shehui*” in Chinese. In fact, the three different Chinese terms do not have the same meaning. Many scholars actually use these different Chinese terms of civil society simultaneously. “*Minjian shehui*” is used in this article because the term is widely used in the academic research in China’s modern civil organizations. “Civil society” in this article refers to a sphere that is conceptually separable from the state and government.

4 At the same time the State Council abolished the *Jijin guanli banfa* stipulated in 1988.

5 The Chinese and English versions of the revised regulation (2016) can be found in Chinalawinfo. “Regulation on Registration and Administration of Social Organizations (2016) (*Shehui tuanti dengji guanli tiaoli*).

6 Nevertheless, grassroots NGOs registered under the MOIC also encounter problems with regard to the amount and source of funding. As registered commercial entities, grassroots NGOs are required to pay a 5 % tax on any revenue, even for funding received for non-profit purposes.

7 In recent years, the abovementioned legislative hurdle in NGO registration has been relaxed in some parts of China, first in Shenzhen and then in Beijing. The local government in these two cities relaxed its NGO registration rule, and allowed NGOs in the fields of business, charity, welfare, and social services to register directly with the MOCA. Such a move can also be observed at the national level. In early 2011, the national government started planning to revise the regulations on NGO registration and management. If the revised version is passed by the State Council, the individual-organized or grassroots NGOs can directly register with the MOCA without seeking a supervisory body prior to the registration.

8 It is noted that the author is fully aware of the arguments put forward by Timothy Hildebrandt, stating Chinese NGOs could be favored or cracked down by the state apparatus irrespective of registration status. The author is argued that unregistered NGOs are comparatively easier to be targeted and cracked down by the government officials in the name of “unlawful organizations”. For Hildebrandt’s argument, see Hildebrandt, T. The Political Economy of Social Organization Registration in China. The China Quarterly. 2011;208:970–89.

9 GONGOs are composed of three types of organizations existed in the 1950s and 1960s: (1) private organizations from the “old China”; (2) friendship associations for the promotion of trade, and cultural exchange agencies; and (3) people’s organizations and mass organizations.

10 In the course of the meeting, US Vice President Al Gore claimed that HIV/AIDS was a security threat because “It threatens not just individual citizens, but the very institutions that define and defend the character of a society. The disease weakens workforces and saps economic strength. HIV/AIDS strikes at teachers, and denies education to their students. It strikes at the military, and subverts the forces of order and peacekeeping”.

11 McInnes and Rushton (2010) argued that the HIV/AIDS securitization in 2000 was not a successful or complete securitization. However, this article does not intend to examine whether the international HIV/AIDS securitization has been fully accepted by the target audience. It intends to examine the influences of the HIV/AIDS international securitization on the national and sub-national levels in China.

12 In 2003, the Bush Administration launched the President’s Plan for Emergency AIDS Relief (PEPFAR), pledging US$15 billion from 2003 to 2008 to “turn the tide against HIV/AIDS.” The WHO launched the “3 × 5” campaign that targets getting 3 million people on antiretroviral treatment by 2005, while the Multi-Country HIV/AIDS Program of the World Bank provided more than US$1.3 billion for grants and concessional loans to help governments respond to HIV/AIDS issues.

13 Examples included the Indian Network of People Living with HIV/AIDS, Thai Network of People Living with HIV/AIDS, Asia Pacific Council of AIDS Service Organization in Malaysia, and also Action for AIDS Singapore,
